# The effect of probiotics used as a single therapy on functional constipation: Study protocol for a systematic review and meta-analysis

**DOI:** 10.1097/MD.0000000000019824

**Published:** 2020-04-24

**Authors:** Yong Wen, Jun Li, Xuegui Tang

**Affiliations:** a Department of Traditional Chinese Medicine, The affiliated Hospital of Southwest Medical University, Luzhou; b Department of integrated Traditional and Western Medicine Anorectal, Affiliated Hospital of North Sichuan Medical College, Nanchong, Sichuan, China.

**Keywords:** constipation, meta-analysis, probiotics, single therapy

## Abstract

**Background:**

Constipation is a frequent gastrointestinal symptom. It is intimately related to many diseases. 1st-line therapy can not alleviate constipation for some patients. Alternative treatments are therefore commonly used, such as probiotics. Nevertheless, the efficacy and safety of probiotics used as a single treatment are still uncertain. A systematic review and meta-analysis will be carried out to answer the issue.

**Methods:**

The protocol accompanied Preferred Reporting Items for Systematic Reviews and Protocol Meta-Analysis. PubMed, Cochrane, Embase, and Web of Science databases were practiced for randomized controlled trials without language constraint. In addition, We have also conducted backward (manually) and forward (with Google Scholar) citation checks to identify any additional relevant papers.

Two reviewers will conduct studies selection, data extraction, and risk of bias assessment independently. The primary outcome is treatment success (spontaneous bowel movements (sBMs) >3 times per week), defecation frequency. The second result will be consistency, fecal incontinence, other symptoms (e.g. flatulence, abdominal pain), and adverse event rates and types.

**Results:**

This study provides helpful information about whether probiotics can be used as a single therapy on functional constipation

**Conclusion:**

The findings of the review will be disseminated through peer-review publications

## 1 Introduction

Constipation is a common gastrointestinal symptom. Constipation is not only the difficulty of defecation or hard defecation but also a series of symptoms, such as straining, flatulence, abdominal discomfort, abdominal pain and spending a long time on the toilet without defecation. The average prevalence of constipation among all adults in the U.S.community is 16%, compared with 33.5% among the elderly (60–101 years old).^[1]^ In addition to the discomfort of constipation itself, constipation is closely related to many diseases such as kidney disease,^[2]^ Parkinson disease,^[3]^ and colorectal cancer.^[4]^ Recent studies have found that constipation and laxative use are independently associated with a high risk of all-cause mortality, coronary heart disease, and ischemic stroke events.^[5]^ In addition, due to the chronic course of the disease and high recurrence rate, constipation consumes a lot of healthcare resources, and the direct annual cost related to constipation management per patient varies from $1912 to $7522 in the United States.^[6]^ Therefore, highlighting the importance of effective prevention and treatment of functional constipation. At present, there are many treatment options on functional constipation, from diet intervention and behavior intervention to drug treatment and surgical choice, however, 49% of the patients who initially received over-the-counter treatment and 58% of the patients who initially received prescription treatment failed in the end.^[7]^ Another study reported that almost half of the respondents were not completely satisfied with their current treatment plan for constipation.^[8]^

In recent years, the application of probiotics in constipation has gradually increased. However, there is no consensus on the effectiveness and safety of probiotics so far.^[9–11]^ In addition to the characteristics of participant and probiotics, how to use probiotics in the treatment of constipation is an important and easily overlooked problem. At present, there are 2 modes of probiotics interventions, 1 is to use them as a single therapy,^[12–13]^ the other is to use them as a co-adjuvant therapy with treatment as usual (TAU).^[14]^ Obviously, compared with the latter, probiotics used as a single therapy have lower healthcare costs and fewer potential adverse drug reactions because it reduces the laxative use. Therefore, we should pay more attention to the efficacy and safety of probiotics used as a single therapy in the treatment of constipation. However, in recent meta-analysis, little attention has been paid to this difference, and the data of 2 different kinds of interventions are combined for analysis, which reduces the reliability of the conclusion and brings confusion to the clinical application,^[15–16]^ Only 1 meta-analysis that evaluate the effectiveness of probiotics on constipation in children mentions this problem.^[17]^

This paper will review and analyze the clinical research of probiotics, focusing on the effectiveness and safety of probiotics used as a single therapy on constipation, and the effect of strain specificity will be focused on as different strains show different therapeutic effects.

## 2 Methods

### 2.1 Registration

The DOI is 10.17605/OSF.IO/X6P9B and has been registered in the OSF. We will use RevMan 5.3 software to build meta-analysis and will make use of the Cochrane Guide for Systematic Reviews of Interventions.^[18]^ We shall report on its results in accordance with the statement of the Preferred reports for systemic reviews and meta-analysis (PRISMA).^[19]^ This research does not need an ethical argument since human intervention is not directly involved.

### 2.2 Eligibility criteria

PICOS approach was used to summarize the eligibility criteria (patients, intervention, comparisons, outcome, and study design type).

#### 2.2.1 Types of participants

Age, ethnic distribution and gender are not restricted for all participants diagnosed with functional constipation according to the Roma III or IV diagnostic criteria. Exclusion criteria including:

1. patients with organic diseases related to digestive tract;
2. patients with constipation induced by drugs;
3. patients with systemic diseases related to constipation such as metabolic and neuropathic diseases.

#### 2.2.2 Comparisons and interventions

Probiotics are given to the treatment group and placebo or another treatment has been compared with probiotics. All probiotics (single or mixture) strains, doses, treatment regimens, and means of administration (tablets, capsules, powder, or fortified foods) will be considered.

#### 2.2.3 Outcome measures

The main result is the frequency of defecation, successful treatment (spontaneous bowel movements (sBMs) >3 times per week). The 2nd outcome is stool consistency, flatulence, abdominal pain, fecal incontinence, and the rates and types of adverse events.

#### 2.2.4 Study types

Only RCTs were eligible for inclusion up to September 1, 2019. There is no language restriction.

### 2.3 Methods for searching

Searches on databases PubMed, Cochrane, Embase and Web of Science were performed to September 1, 2019 with no restriction of languages. We also carried out backward (manually) and forward (with Google Scholar) citation checks in order to identify any further relevant papers, by reviewing the reference lists of previous systematic reviews on the subject, and all of the relevant documents chosen from the bibliographic databases search as well as all subsequent publications citing them. The search strategy will include the following search terms: constipation, probiotics, randomized controlled trial. A detailed search strategy is described in Table 1.

**Table 1:**
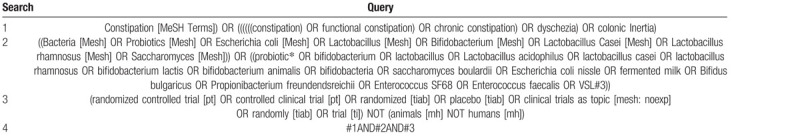
Preliminary search strategy in PubMed.

### 2.4 Selection of studies and extraction of data

#### 2.4.1 Study selection

**Figure 1:**
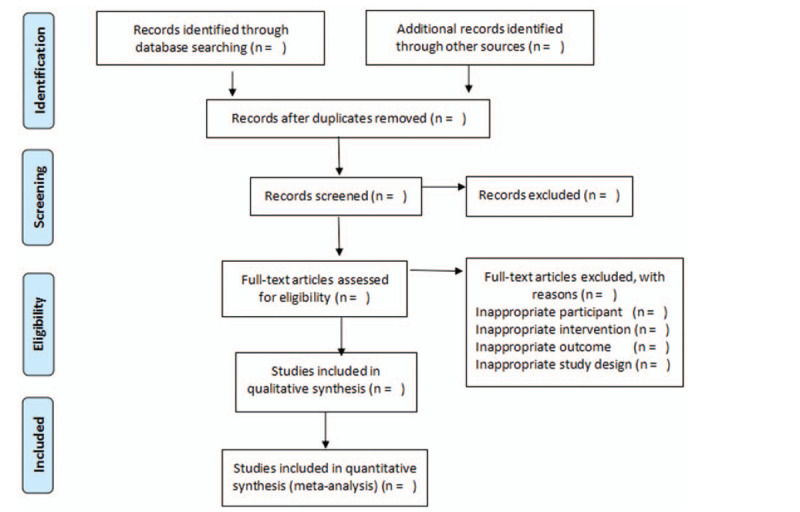
Flow diagram of study selection.

After electronic scans of the databases, eliminating duplicate findings, 2 reviewers will independently scan the titles and abstracts to find studies that might meet the inclusion criteria outlined above. The 2 review authors will retrieve the full text of these potentially eligible studies and independently evaluate them for eligibility. Any discrepancy will be resolved by consensus or judged by a third reviewer. The selection process is illustrated with a PRISMA flow diagram (Fig. 1).

#### 2.4.2 Data extraction

Relevant data will be extracted independently from the studies. No matter the research, relevant data will be collected. The data will include age of patients, sex, number of patients randomized to either probiotics or control interventions, diagnostic criteria, methods, settings, type of interventions (probiotic strain(s) and species, doses, intervention length), primary, and secondary consequences examined. Outcomes include defecation frequency and the number of patients that achieved treatment success (spontaneous bowel movements (sBMs) >3 times per week), stool consistency, other symptoms (e.g., flatulence, abdominal pain), fecal incontinence, and the rates and types of adverse events. We will contact study authors to request for any data missing or clarifications needed. For the recording of information, a unified form of data extraction was used. First, 2 reviewers have extracted all the data, and then the data is verified for accuracy. A third reviewer resolved disagreements.

### 2.5 Risk of bias assessment

The tool for evaluating the risk of bias used by Cochrane Collaboration was to assess the risk of bias.^[18]^ Two authors will check the contents as follows: random sequence generation and concealment of allocation (selection bias), blinding of participants and personnel (performance bias), blinding of outcome assessment (detection bias), incomplete outcome data (attrition bias), selective reporting (reporting bias), and other sources of bias.

As per the recommendations of the Cochrane Handbook, each item was scored as at low (+)or high (−) risk of bias depending on whether each studys methods fulfilled or did not fulfill these criteria, judgment will be recorded as unclear (?) risk of bias if information about these factors did not appear in the publication. Disagreements will be solved by discussion, with involvement of a 3rd author.

### 2.6 Data synthesis and statistical analysis

#### 2.6.1 Data synthesis

We will develop the meta-analysis using RevMan 5.3 software. Dichotomous outcomes such as treatment success will be recorded as risk ratios (RRs) with a confidence interval (CI) of 95%. For a continuous variable, the mean difference, and 95% CI are calculated. When the method or unit of measurement of the effect of the same intervention is identical, weighted average difference (WMD) is best selected. When various measuring instruments or units are used with the same intervention effect, or when the mean difference between different experiments are too large, standard mean difference (SMD) will be used as the composite statistics. *P* < .05 is regarded as indicating statistical significance.

#### 2.6.2 Assessment of heterogeneity

Heterogeneity will be assessed by the Chi-squared test and the *I*^2^ test, with a value 50% considered to represent substantial heterogeneity. The heterogeneity is acceptable when *I*^2^ < 50%, and a fixed impact model is used for statistical analysis. Otherwise, if *I*^2^ is equal or over 50%, a model of random effects will be used to analyze data and then we will use sensitivity analyzes to investigate possible justifications. To show significance a *P* value of .05 was considered.

#### 2.6.3 Sensitivity analysis

To check the reliability of the pooled data, we will remove every single study in turn and replicate the meta-analysis. Subgroup research was conducted where a particular species or strain had ample trials.

### 2.7 Assessment of reporting bias

If more than 10 of the studied grapes are included, a funnel plot regression will be evaluated. We will investigate the (a)symmetry in (inverted) funnel plots, which show the association between effect size and accuracy of study.

### 2.8 Confidence in cumulative evidence

The quality of evidence will be assessed on the GRADE (Grading of Recommendations Review, Production, and Evaluation) framework foundation. It will segregate the scientific evidence into 4 levels: high, moderate, low, and very low.

## 3 Discussion

At this point, Probiotics is still controversial in the treatment of functional constipation, in addition to the characteristics of probiotics (e.g. isolation sources, doses, and duration of treatment) and participant characteristics, we also need to consider how to use probiotics. Therefore, this study aims to determine whether probiotics used as a single therapy can effectively treat constipation and provide reasonable suggestions for the rational use of probiotics in the treatment of constipation.

## Author contributions

**Conceptualization:** Yong Wen.

**Data curation:** Yong Wen, Jun Li.

**Methodology:** Yong Wen, Jun Li.

**Project administration:** Yong Wen.

**Supervision:** Xuegui Tang.

**Writing – original draft:** Yong Wen.

**Writing – review & editing:** Yong Wen, Xuegui Tang.

Yong Wen orcid: 0000-0002-2538-0463.
